# Multiple Players Tracking in Virtual Reality: Influence of Soccer Specific Trajectories and Relationship With Gaze Activity

**DOI:** 10.3389/fpsyg.2022.901438

**Published:** 2022-05-20

**Authors:** Alexandre Vu, Anthony Sorel, Annabelle Limballe, Benoit Bideau, Richard Kulpa

**Affiliations:** Univ Rennes, Inria, M2S – EA 7470, Rennes, France

**Keywords:** visual exploration, attention, soccer (football), virtual reality, visual tracking task, eye-tracking

## Abstract

The perceptual-cognitive ability to track multiple moving objects and its contribution to team sports performance has traditionally been studied in the laboratory under non-sports specific conditions. It is thus questionable whether the measured visual tracking performance and the underlying gaze activity reflected the actual ability of team sports players to track teammates and opponents on a real field. Using a Virtual Reality-based visual tracking task, the ability of participants to track multiple moving virtual players as they would do on a soccer field was observed to pursue two objectives. (i) See the influence of different scenario types (soccer-specific trajectories versus pseudo-random trajectories) on the visual tracking performance of soccer (*n* = 15) compared to non-soccer players (*n* = 16). (ii) Observe the influence of spatial features of the simulated situations on gaze activity between soccer players and non-soccer players. (i) The linear mixed model regression revealed a significant main effect of the group but no interaction effect between group and the type of trajectories, suggesting that the visual tracking ability of soccer players did not benefit from their specific knowledge when they faced scenarios with real game trajectories. (ii) Virtual players’ spatial dispersion and crowding affected the participants’ gaze activity and their visual tracking performance. Furthermore, the gaze activity of soccer players differed in some aspects from the gaze activity of non-soccer players. Assumptions are formulated as to the implication of these results in the difference in visual tracking performance between soccer players and non-soccer players. Overall, using soccer-specific trajectories might not be enough to replicate the representativeness of the field conditions in the study of visual tracking performance. Multitasking constraints should be considered along with motor-cognitive dual-tasks in future research to develop the representativeness of visual exploration conditions.

## Introduction

Soccer players continuously monitor the position and behavior of several teammates and opponents in order to act properly when the time comes. These sources of information are scattered over a field of about 100 m long by 60 m wide but, at a given time, one can pay attention only to a limited part of the environment (Memmert, 2009). Skilled soccer players demonstrated higher perceptual-cognitive abilities to overcome these constraints and properly allocate their visual attention in this complex and dynamic environment (Williams and Davids, 1998; Vaeyens et al., 2007; Roca et al., 2013). However, the underlying mechanisms of the ability to visually track the movement of multiple teammates and opponents over time are not all well known (Vater et al., 2017; Qiu et al., 2018; Harris et al., 2020a; Jin et al., 2020).

The relationship between sports performance and visual tracking ability (e.g., Memmert et al., 2009; Faubert, 2013; Mangine et al., 2014) was studied in the laboratory with the Multiple Object Tracking (MOT) task (Pylyshyn and Storm, 1988; Meyerhoff et al., 2017). This MOT task was used to study some fundamental perceptual-cognitive abilities related to sports performance (Scharfen and Memmert, 2019). This task reflects the attentional demands of soccer in terms of the dynamic distribution of the attention over multiple moving sources of information (e.g., teammates and opponents). In this task, multiple target objects must be tracked for several seconds in a space shared by disruptive objects of the same appearance. Observers must correctly allocate their attentional resources toward the targets to avoid loss or confusion with a disruptor (Drew et al., 2013). Previous studies suggested that the number of targets that can be successfully tracked is limited (Tombu and Seiffert, 2008), and depends on several situational features such as the number of targets to track (Pylyshyn and Storm, 1988), their spatial dispersion (Shim et al., 2008), the number of disruptors to abstract (Bettencourt and Somers, 2009), their density around the targets (Iordanescu et al., 2009), or the speed of objects (Tombu and Seiffert, 2011).

The tracking ability also depends on the intrinsic perceptual-cognitive abilities of the observers. Indeed, differences in visual tracking performance was observed in the laboratory study by Allen and colleagues between a group of aircraft radar operators and a control group (2004). The advantage of the operators group was assumed to be related to their daily activity which induces the tracking of several points on a digital screen, i.e., attentional demands similar to those of the MOT task (Allen et al., 2004). An expertise effect has also been observed with action video game players compared to non-video games players (Green and Bavelier, 2006). Moreover, non-gamers demonstrated increased visual tracking performance after regular training with action video games (Green and Bavelier, 2006). This evidence suggests that visual attention measured in the visual tracking task increases when regularly stimulated by an activity such as action video games (Green and Bavelier, 2006). In relation to the sports domain, inter-individual differences were also found between practitioners and non-practitioners of team sports (Qiu et al., 2018; Harris et al., 2020a; Jin et al., 2020). However, this effect seemed to be moderated by the level of practice (Faubert, 2013; Qiu et al., 2018) and the players position on the field (Mangine et al., 2014; Martín et al., 2017). The study by Memmert et al. (2009) was the only one to not find any differences between groups on an MOT task. This result may be partly justified by a too low level of difficulty in the proposed task (Qiu et al., 2018). Also, Qiu and colleagues suggested that the development of visual tracking abilities does not evolve linearly with sport performance (2018). Even if handball players in the study by Memmert and colleagues practiced for more than 10 years, they may not have been playing at a level that allowed them to develop visual tracking abilities. Another explanation may be the lack of correspondence between the attentional constraints of the MOT task and those of the field. Memmert and colleagues speculated that inter-individual differences might have been observed with an assessment task that draws on more domain-specific aspects of expertise (Memmert et al., 2009). Meyerhoff and colleagues’ introductory review on MOT emphasized the importance of incorporating more natural, real-world visual tracking scenarios to support the ecological validity in the study of sports performance (2017).

The ecological validity issue has also been addressed concerning the benefits’ transfer of perceptual-cognitive training to an actual sport-specific task (Hadlow et al., 2018; Walton et al., 2018; Vater et al., 2021). Despite initial promising results observing a transfer from visual tracking training to improved passing decision making in soccer (Romeas et al., 2016), contradictory results were repeatedly observed (Fleddermann et al., 2019; Harris et al., 2020b; Harenberg et al., 2021). Limited transfer to real-world performance may be due to a mismatch between the displayed stimuli or between the observation conditions (Hadlow et al., 2018). To this end, Ehmann et al. (2021, 2022) proposed a 360° alternative of this tracking task with humanoid avatars running on a curved screen surrounding the observer. This configuration allowed for attentional constraints closer to the field; i.e., to-be-tracked sources of information that move all around an observer and not just on a frontal plane. The authors were able to validate the use of their tool to assess visuo-spatial performance in a visual tracking task (Ehmann et al., 2021) and its use to discriminate the effect of expertise in young soccer players (Ehmann et al., 2022).

However, to our knowledge, no investigation focused on the use of sport-specific trajectories to animate the objects to perceive in a visual tracking task. The use of sport-specific trajectories would be another way to increase the correspondence of stimuli to real-world situations (Hadlow et al., 2018). With real game trajectories, structured collective behaviors may emerge from relative movements between virtual players (North et al., 2009). The perception of structured collective movements, allowed by the use of sport-specific knowledge, seemed to be used by soccer players to anticipate the outcome of game situations (North et al., 2016). Perception of structured collective movements was still possible with only the relative position of players on the field, without additional discerning features (e.g., body orientation and posture) (North et al., 2016). Therefore, the visual tracking ability of soccer players could also benefit from specific knowledge when visualizing scenarios with soccer-specific trajectories. This advantage would disappear in random movement scenarios (Williams and Davids, 1995; North and Williams, 2019).

Visual tracking performance appeared to be associated with different cognitive processes such as visuospatial attention and working memory (Harris et al., 2020a; Ehmann et al., 2021), but also with effective gaze control (Hyönä et al., 2019). This gaze control aimed to combine different attentional strategies in order to meet the constraints of the MOT task. Eye-tracking measurements in laboratory MOT tasks have shown that gaze fixations alternate between individual targets and a location in the center of them (Fehd and Seiffert, 2008, 2010; Zelinsky and Neider, 2008). Looking overtly at a target allows detailed processing of its information to differentiate it from the rest of the environment. The fixation of a central point allows to minimize the retinal eccentricity of each target and to track them by avoiding the suppression of visual information related to saccades. This central point, i.e., the centroid, is usually defined as the center of mass of the objects to be monitored, but it may be closer to crowded areas in order to reduce the negative influence of visual interference between targets and nearby disruptors (Lukavský, 2013; Vater et al., 2017). Looking at the centroid has a positive influence on visual tracking accuracy, but saccades to individual targets are still necessary either when their resolution in the periphery does not allow reliable processing of visual information (Fehd and Seiffert, 2010), or when they are about to be occluded (Zelinsky and Todor, 2010; Kamkar et al., 2020), or when they change direction after collision (Vater et al., 2017).

The shift of fixations between the centroid and individual targets is called a “center-target switching strategy” in the MOT literature (Fehd and Seiffert, 2010). This is similar to the “visual pivot” strategy in the sports science literature, defined as a behavior where gaze “is shifted frequently between different cues around a pivot location” (Vater et al., 2020). The fixed location used as a visual pivot minimizes the distance to peripheral objects and thus limits the suppression of visual information during saccades to these objects (Klostermann et al., 2020; Vater et al., 2020). This strategy is linked to another strategy called “gaze anchoring” strategy, defined “as the use of a rather long fixation on a position that is not necessarily related to an informative cue but that optimizes information processing via peripheral vision and eliminates the costs associated with saccadic eye-movements” (Vater et al., 2020). Using eye tracking measurements (Kredel et al., 2017), visual pivot can be differentiated from gaze anchoring with a higher number of shorter fixations (Vater et al., 2020). These two strategies are not exclusive (Klostermann et al., 2020). Nevertheless, the visual pivot seems preferable in situations with low temporal pressure to support the cost of the saccade. Conversely, visual anchoring seems preferable in situations with high temporal pressure to avoid saccadic suppression (Vater et al., 2020).

In MOT tasks, increasing object speed induced a reduction in the number of saccades, a reduction in target fixation time but an increase in center fixation time (Hyönä et al., 2019). Because the accuracy of visual tracking depends on gaze activity (Fehd and Seiffert, 2010), appropriate gaze behaviors in visual tracking tasks are expected in individuals who are regularly faced with similar attentional constraints, e.g., team sports players who have to monitor multiple teammates and opponents over time. Different visual search activities were observed between skilled and less skilled soccer players during a decision making task when viewing videos of soccer situations (Roca et al., 2013). In addition, more adaptations to the constraints of the visualized situations (high time pressure versus low time pressure) were observed in skilled soccer players compared to less skilled soccer players (Roca et al., 2013). However, there is very little data on the effect of expertise on gaze activity during a visual tracking task. In the study by Harris and colleagues, no differences in gaze activities were observed during a MOT task between team sports players and non-team sports players (2020a). The authors argued that the superior visual tracking performance of team sport players relied more on core perceptual-cognitive abilities, i.e., working-memory, than on a specific control of overt attention (Harris et al., 2020a). However, in their study, participants tracked objects that moved on a 2D screen in front of them (Harris et al., 2020a). The gaze activity underpinning the tracking of moving dots on a 2D screen had probably not undergone the same constraints as the tracking of teammates and opponents on a real field (Ehmann et al., 2021).

Ensuring viewing conditions close to those of the expertise domain would favor the difference in visual search activity between skilled and less skilled athletes (Mann et al., 2007; Klostermann and Moeinirad, 2020). Using virtual reality (VR) seems to be an appropriate solution to overcome this viewing limitation (McGuckian et al., 2018; Rojas-Ferrer et al., 2020). VR devices display a computer-generated immersive virtual environment in stereoscopy, which allows an observer to access depth cues that have been relevant for tracking multiple objects (Cooke et al., 2017). In addition, the adaptive first person viewpoint enhances the sense of *presence* in the environment, i.e., the feeling of actually belonging to the virtual world (Loomis et al., 1999; Slater and Sanchez-Vives, 2016). This sense of *presence* is crucial to act naturally as on a field, both to perform specific and technical gestures (Brault et al., 2012; Vignais et al., 2015) but also to carry out visual exploration behaviors that guide performance in sport-specific perceptual-cognitive task (Rojas-Ferrer et al., 2020). Under these 3D conditions, gaze activity results not only from eye movements, but also from body and head adjustments (McGuckian et al., 2018; Ehmann et al., 2021).

To address the concerns raised above in the investigation of visual tracking performance, i.e., the lack of sport-specific scenarios and the lack of representative visual exploratory conditions, this study proposed a multiple soccer players tracking task in VR. The primary objective was to compare the visual tracking performance of soccer players and non-soccer players perceiving virtual players moving along real game or pseudo-random trajectories. It was expected to see an interaction effect, such that visual tracking performance between soccer players and non-soccer players was higher when viewing soccer-specific scenarios than when viewing pseudo random scenarios (Williams and Davids, 1995; North and Williams, 2019). The second objective of this study was to observe the influence of the spatial features of situations on the gaze activity of soccer players and non-soccer players. Different gaze activities were expected between situations according to the spatial distribution of virtual players (Vater et al., 2017). And, most interestingly here, different gaze activities were expected between soccer players and non-soccer players during the proposed visual tracking task (Vater et al., 2020).

## Materials and Methods

### Participants

Thirty-three volunteers with normal or corrected-to-normal vision completed the experiment. Two participants were removed from the data analysis because 20% of their trials were invalid due to failure in applying the target selection instruction (see section “Visual Tracking Task”). At the end, 16 participants (24.3 years old ± 3.4, 3 women – 13 men) who had been playing soccer in competition for an average of 15.6 years (± 3.9) for 6.6 h per week (± 2.5) at the time of the experiment were included in the SOCCER group. Four participants were playing at district level, 9 at regional level and 3 at the French national level. Fifteen participants (28.3 years old ± 4.6, 8 women – 7 men) who did not practice any team sport in competition joined the CONTROL group. In accordance with the Declaration of Helsinki, written consent was obtained prior to participation. The experimental protocol and the data collection procedure were validated beforehand by a French national ethics committee in sports sciences (IRB00012476-2021-16-04-102).

### Virtual Soccer Players Tracking Task

**FIGURE 1:**
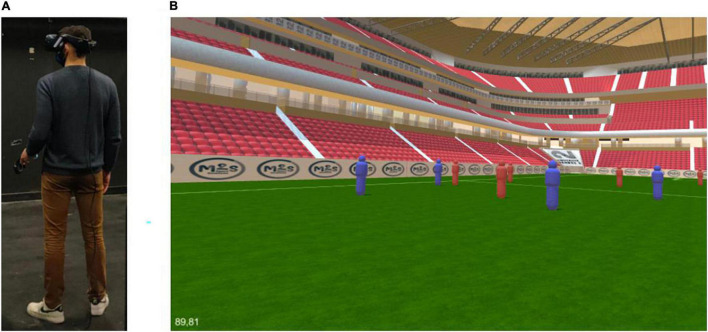
**(A)** Experimental layout with a participant standing in the real world with the VR-HMD and the joystick controller. **(B)** The view of the virtual world projected in the VR-HMD.

Participants wore a VR Head Mounted Display (VR-HMD) in a standing position (Figure 1A). In the visual tracking task, the participant took the vantage point of a central defender on a soccer field and visualized situations with 10 virtual moving teammates in blue and 11 opponents in red (Figure 1B). The tracking task was processed as follows. At the beginning of the trial, all virtual players were stationary and four arrows indicated the to-be-tracked target-players. When target-players were correctly identified, the participant pressed the corresponding button on the joystick to start the trial. From this moment, the arrows disappeared and it was no longer possible to visually distinguish the target-players from the other virtual players which were now considered as disruptors. After 2 s, all the virtual players started to move for 10 s. At the end of the moving phase, the virtual players stopped and the participant had to point with the joystick the four target-players. The participant’s responses and the gaze direction over time were recorded at each trial.

### Procedure

Participants completed 30 trials in total. Of these, 15 trials were performed in a so-called structured experimental condition (STRU), presenting real soccer games scenarios, which therefore included structured collective movements between the virtual players. The other 15 trials were conducted in a so-called unstructured experimental condition (UNSTRU), presenting pseudo-random movement scenarios with no collective structure in the relative movement of the virtual players. These trials were presented to the participants in a random order. Prior to the evaluation, participants underwent an eye-tracking calibration and a training phase of four trials to familiarize themselves with the virtual environment and the proposed tracking task. Only the results of the evaluation phase were analyzed. The total duration of the tracking task with the VR-HMD was approximately 35 min.

### Apparatus

#### Hardwares and Softwares

The VR-HMD used with an integrated eye tracker was an HTC VIVE EYE PRO. The recording frequency of the integrated Tobii eye tracker was 90 Hz. According to the hardware specifications^1^ the margin of error of the eye tracker was 1.1° and the calibration was a five-point procedure. The display resolution in the HMD was 1,440 × 1,600 pixels per eye (2,880 × 1,600 pixels combined) with a total field of view of 110°. The capture space of the HMD used was 2 × 2 m, allowing participants to adjust their position and orientation during the tracking task (Figure 1). An HTC Vive Controller was used as a joystick by the participants to process the different phases of the tracking task. The VR-HMD was connected to a Dell Precision 5,820 computer with a processor Intel(R) Xeon(R) 3.60 GHz and a graphics card NVIDIA GeForce RTX 2080 Ti ensuring high fluidity in the display of the immersive virtual environment (90 Hz). The environment was developed with the Unity 3D game engine (editor’s version 2019.4) and was built in a standalone mode. The SRapinal SDK for Unity was used to access the gaze data recorded by the eye tracker. The MiddleVR (version 1.7.3) middleware was used to interface the HTC VIVE hardwares with the Unity build software.

#### Simulated Scenarios

In STRU condition, the real player movement scenarios were simulated from 15 real match situations from a database of 7500 situations (Data Source: STATS, copyright 2019^2^). In UNSTRU condition, the 15 pseudo-random movement scenarios were computer generated from these 15 real situations. The procedure of selection of the real game situations for the STRU condition was as follows. Only situations of at least 10 s were retained. Counter-attack and free-kick situations were removed. Retained open play situations were ordered by the mean speed of both teams, the mean acceleration of both teams, the mean players’ dispersion of both teams and the mean distance between both teams centroid. Finally, 15 situations were selected after a visual inspection by the investigators from a defender vantage point. The procedure of generation of pseudo-random trajectories for the UNSTRU condition was as follows. From each of the 15 real situations, a destructured situation was generated in such a way that starting location, ending location and mean speed of virtual players were similar to the real situation. The path between the starting and ending locations of each virtual player was constrained by an imaginary surrounding box. The virtual players went straight ahead at a constant speed until they “bounced” off a wall of the imaginary box. They kept going straight and bounced until reaching the final location. The initial starting angle was randomly selected from the angles that allowed the virtual players to reach the final location in the desired time. No collective structured behaviors were assumed to emerge from the relative movements of virtual players in UNSTRU situations. The target-players were selected by the investigators so that the target-players were scattered over a maximum range of 90° on each trial.

### Gaze Behaviors Classification

#### Definitions

The analysis of exploration behaviors here was done under the scope of gaze activity in a 3D virtual world rather than under the scope of eye movements in relation to the head (Lappi, 2016). Gaze activity resulted from body, head and eye movements (McGuckian et al., 2018), and ultimately corresponded to the movements of the 3D vector materializing the line of sight between the eyes and the visual target relative to an allocentric reference frame (Lappi, 2016). Thus, the term fixation here referred to a gaze fixation (rather than eye fixation), which could be defined as the fixation of the point of gaze on an object or a location in space (Lappi, 2016). In addition, a saccade was defined here as an overt movement allowing a shift in the point of gaze fixation.

#### Classification Algorithm

**FIGURE 2:**
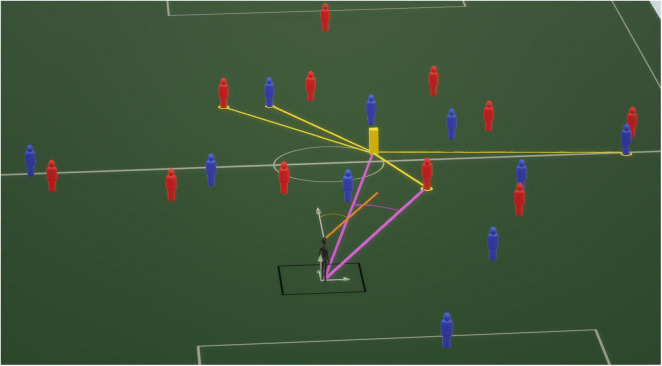
Schema of the experimental configuration in the virtual environment. The black humanoid represents a participant, and the red and blue mannequins represent the virtual players. From this schema, only the soccer field and the virtual players were visible to the participant during the experiment. The black lines represent the allowed moving space for the participant, and the gray arrows represent the reference axis of the virtual 3D space. One axis corresponds to the field length, one to the field width, and the last one to the vertical axis. The orange line corresponds to the participant’s gaze direction. The gaze angle, illustrated by the orange circle arc, was computed as the angle between the gaze direction vector and the field length axis. The yellow brick represents the target players’ centroid, computed as the mean target players’ location. For each target player, an eccentricity angle was computed, e.g., illustrated by the pink circle arc, as the angle between target-to-participant vector and centroid-to-participant vector represented with pink lines. The target-to-centroid angles were used to order the target players according to their distance to the centroid.

A custom Identification-Velocity Threshold (I-VT) based algorithm was used to classify the participants’ gaze behaviors in this study (see Salvucci and Goldberg, 2000 for pseudo-code). The raw data recorded from Unity was the gaze direction in the virtual 3D space. However, the gaze direction relative to the vertical *Y* axis averaged −1.80° with a standard deviation of 1.73°. In other words, the participants were looking constantly at eye level. Therefore, gaze direction on *Y* axis was not taken into account for the classification of gaze behaviors. The gaze direction on the XZ plane, which corresponds to the virtual soccer field plane, was used to calculate the angle of gaze direction (in degrees) around the vertical axis relative to the longitudinal axis of the virtual field (Figure 2, orange line). For different velocity thresholds tested (40–100°), the results of a preprocessing of the I-VT algorithm on the gaze angle of 10 randomly drawn trials were compared to a manual classification of two operators (two investigators of the study). The number of fixations, fixation duration, number of saccades, and saccade amplitude returned by automatic and manual classifications were used as comparison parameters. At the end, the velocity threshold of 60°/s was retained for the whole classification of the gaze dataset. For measure of sample-by-sample agreement (Andersson et al., 2017), Cohen’s Kappa and F1-score revealed a high reliability with operator 1 to classify fixations (Cohen’s Kappa = 0.88 and F1-score = 0.98) and saccades (Cohen’s Kappa = 0.89, F1-score = 0.90). A correct reliability with operator 2 to classify fixations (Cohen’s Kappa = 0.73, F1-score = 0.96) and saccades (Cohen’s Kappa = 0.83, F1-score = 0.85) was observed. Therefore, the classification returned by the I-VT algorithm was assumed with reasonable confidence to be similar to a human classification (considered as ground truth) (Andersson et al., 2017).

### Data Analysis

#### Visual Tracking Performance

The visual tracking performance of the participants was computed as the ratio between the number of successfully tracked target-players and the total number of target-players over all trials per experimental condition.

#### Gaze Activity

Gaze activity was described under a set of exploration variables:

– The search rate was computed as the number of fixations divided by the mean duration on an individual trial. High search rate reflected many short fixations whereas low search rate reflected few fixations of long duration (Harris et al., 2021).
– The amplitude of saccades (degrees) was computed as the mean angular distance between initial and final locations of each saccade.
– The gaze exploration variability (degrees) was computed as the Root Mean Square Error between gaze direction and centroid perceived direction. The centroid perceived direction was calculated as the average direction between the target-players. In Figure 2, centroid is illustrated as the yellow brick at the center of the four target-players. In addition to the amplitude of the saccades, this indicates how far the gaze was from the centroid, which was the theoretical point minimizing the sum of eccentricity of target-players (Hyönä et al., 2019).
– The dwell time (seconds) on each Area of Interest (AOI) was computed as the cumulative duration of fixation on each AOI. The six identified AOIs were the four target-players, the centroid and “other” which corresponded to any other space or object in the virtual environment. Target-players were ranked according to their average proximity to centroid (e.g., purple line in Figure 2). Therefore, R1, R2, R3 and R4 AOIs corresponded, respectively, to the closest target-player to centroid (rank 1), the 2nd closest target-player to centroid (rank 2), the 3rd closest target-player to centroid (rank 3) and the farthest target-player to centroid (rank 4). Target-player or centroid AOI was identified as fixated when distance between gaze direction and AOI perceived direction was under a threshold of 1.5° during the fixation. Otherwise, “other” was identified as fixated if the gaze did not match any of the aforementioned AOI. The dwell time variables provided indications of how long the visual cues were observed during the trial.

For each participant, these exploration variables were computed for each of the 30 trials.

#### Trial Situational Features

The 30 trials were clustered, based on a K-means algorithm, according to the dispersion of the target-players and the crowding of disruptors near the targets. In fact, negative effects of crowding on visual tracking accuracy were reported when the distance between a disruptor and a target was under 3° of visual angle (Iordanescu et al., 2009; Vater et al., 2017). The dispersion of the target players in a trial was computed as the average of the standard deviation of the perceived angle of the four target-players. A high dispersion value means that target-players were fairly scattered during the trial and conversely. The crowding in a trial was computed as the average distance that separates each target from its closest interferer. A low crowding value means disruptors were fairly close to target-players during the trial and conversely. Finally, for each trial, a situation score was computed as the mean number of target-players successfully tracked by the participants. It provided an indicator of the difficulty of the visual tracking task in the trial.

#### Statistical Analysis

A linear mixed-model regression (LMM) was used to observe the between-subjects effect of the group of participants, the within-subjects effect of experimental condition and their interaction effect on visual tracking performance. Successive LMMs were run on search rate, amplitude of saccades and gaze exploration variability to observe the within-subjects effect of the cluster of trials, the between-subjects effect of the group of participants and their interaction effect. LMM was run on dwell time to observe the within-subjects effects of the cluster of situations, the between-subjects effect of the group of participants, the within-subjects effect of AOI and their interaction effects. For each linear mixed model regression, a random intercept was set regarding the repeated measures of each participant. Pairwise comparisons with Tukey adjustment on *p* value were conducted in further analysis when significant main effects were observed. Outliers were identified and discarded when they were more than 3 standard deviations from the mean. Normality and homoscedasticity of residuals were inspected with diagnostic plots. Gaze exploration variability has been log transformed because of the substantial heteroscedasticity in original data. Dwell time has been square root transformed because of the substantial non-normality of the original data. The statistical analysis was run in R (version 4). The “Lme4” package was used to run the regressions (Bates et al., 2015), the “effectsize” package was used to compute the effect size (partial eta squared) of each main effect (Ben-Shachar et al., 2020), the “emmeans” package was used for pairwise comparisons in further analysis (Lenth et al., 2020), the “rstatix” package was used to run the K-mean clustering (Kassambara, 2020) and the “fviz_nbclust” function from “factoextra” package was used to define the number of clusters in K-means algorithm (Kassambara and Mundt, 2020).

Occasionally, participants unintentionally terminated a trial without selecting four virtual players. These trials were considered invalid and were removed from the data analysis. In this regard, among the 30 trials for the 31 participants, 14 invalid trials have been removed for the analysis of visual tracking performance (either 1.5% of the overall data). Regarding the gaze activity analysis, an individual trial has been removed in addition because no saccade was measured. After a visual inspection of this individual trial, it was assumed that too much noise in the signal did not allow a reliable classification of gaze behavior. Therefore, 1.6% of the overall data was discarded for the gaze activity analysis.

## Results

### Visual Tracking Performance

**FIGURE 3:**
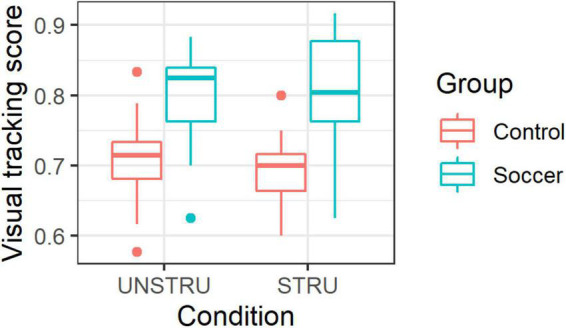
Visual tracking performance by group in both experimental conditions.

Groups’ performance by condition are presented in Figure 3. The LMM on visual tracking performance revealed a significant main effect of group [*F*(1,29) = 21.510, *p* < 0.001, *η_*p*_^2^* = 0.43], with higher visual tracking performance in soccer players (0.804 ± 0.072) than in non-soccer players (0.703 ± 0.060), but no significant main effect of condition [*F*(1,29) = 0.037, *p* = 0.848, *η_*p*_^2^* = 0.01] and no significant interaction effect [*F*(1,29) = 1.369, *p* = 0.252, *η_*p*_^2^* = 0.05].

### Gaze Activity

#### Cluster of Trial Definition

**FIGURE 4:**
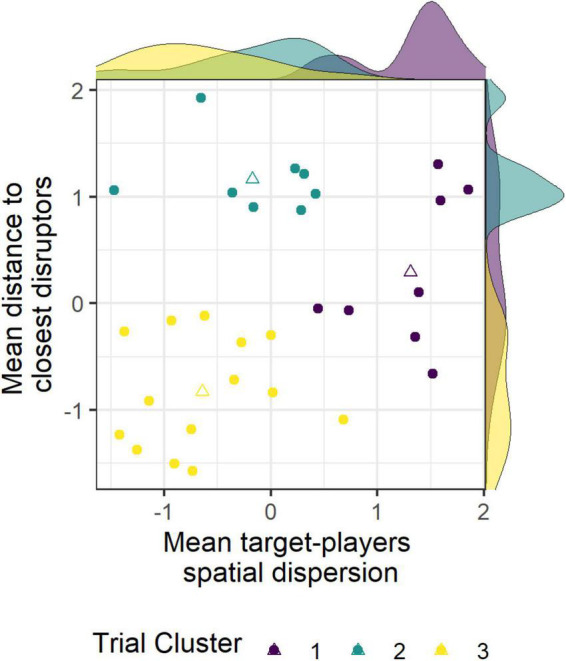
Trials clustering with K-means in three clusters (purple, blue, and yellow, respectively) based on mean target-players spatial dispersion and mean distance to closest disruptors. Both variables were centered and reduced. Triangles represent the mean value of each cluster.

Three clusters of trials were obtained using the K-means algorithm based on the mean target-players spatial dispersion and the mean distance to closest distractors variables (Figure 4). Cluster #1 was composed of eight trials, purple dots in Figure 4, with high mean spatial dispersion of target-players and medium-to-high mean distance to closest distractors. Cluster #2 was composed of eight trials, blue dots in Figure 4, with low mean spatial dispersion of target-players and high mean distance to closest distractors. Finally, cluster #3 was composed of 14 trials, yellow dots in Figure 4, with low mean spatial dispersion of target-players and with low mean distance to closest distractors. The exploration variables were average for each cluster of trials.

#### Search Rate

**FIGURE 5:**
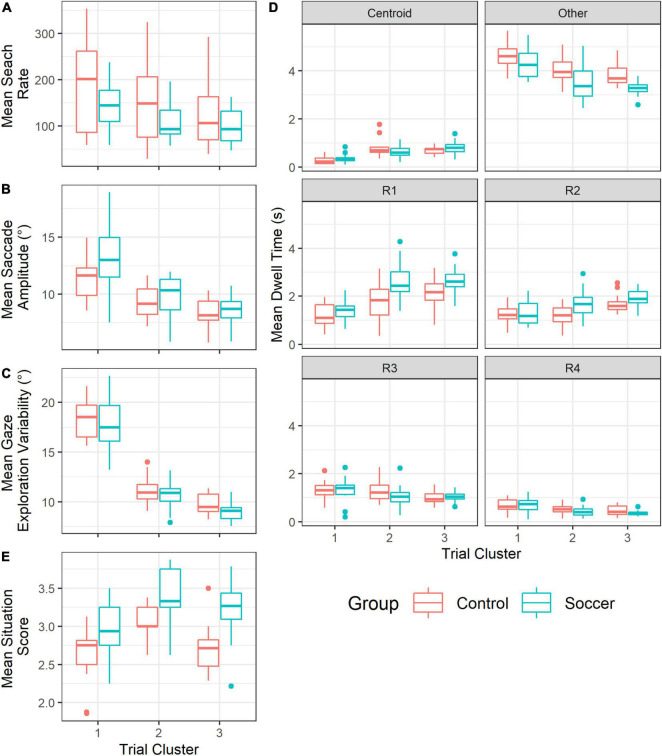
Visual exploration variable per group (Soccer in blue and Control in red) and per trial cluster (1, 2 and 3, respectively). **(A)** Mean search rate, **(B)** Mean saccade amplitude (°), **(C)** Mean gaze exploration variability (°), **(D)** Mean dwell time (s) per AOI, and **(E)** Mean situation score.

The LMM revealed a significant fixed effect of trial cluster [*F*(2,58) = 26.26, *p* < 0.001, *η_*p*_^2^* = 0.48], but no significant fixed effect of group [*F*(1,29) = 2.656, *p* = 0.114, *η_*p*_^2^* = 0.08], and no significant interaction effect between trial cluster and group [*F*(2,58) = 1.076, *p* = 0.348, *η_*p*_^2^* = 0.04] on mean search rate. The further analysis revealed (Figure 5A) a lower search rate (*p* < 0.001) in cluster #2 (127.123 ± 67.201) than in cluster #1 (166.960 ± 86.212), a lower search rate (*p* < 0.001) in cluster #3 (110.864 ± 54.288) than in cluster #1 but no difference between cluster #2 and cluster #3 (*p* = 0.104).

#### Saccade Amplitude

The LMM revealed no significant fixed effect of group [*F*(1,29) = 2.004, *p* = 0.167, *η_*p*_^2^* = 0.06], but a significant fixed effect of trial cluster [*F*(2,58) = 117.163, *p* < 0.001, *η_*p*_^2^* = 0.80], and a significant interaction effect between trial cluster and group [*F*(2,58) = 3.731, *p* = 0.030, *η_*p*_^2^* = 0.11] on mean saccade amplitude. The further analysis revealed (Figure 5B) a saccade amplitude higher (*p* = 0.016) in soccer players (13.085 ± 2.697 degrees) than in non-soccer players (11.488 ± 1.829 degrees) for trials of cluster #1, but no difference (*p* = 0.393) between soccer players (9.852 ± 1.746 degrees) and non-soccer players (9.302 ± 1.384 degrees) for trials of cluster #2 (*p* = 0.393) and no difference between soccer players (8.543 ± 1.231 degrees) and non-soccer players (8.230 ± 1.285 degrees) for trials of cluster #3 (*p* = 0.701). Also, the further analysis revealed for both group a lower saccade amplitude (*p* < 0.001 for both group) in trials of cluster #2 (9.586 ± 2.420 degrees) than in trials of cluster #1 (12.312 ± 1.580 degrees), a lower saccade amplitude (*p* < 0.001 for both group) in trials of cluster #3 (8.423 ± 1.242 degrees) than in trials of cluster #1 and a lower saccade amplitude in cluster #3 than in cluster #2 (*p* = 0.024 for non-soccer players and *p* = 0.002 for soccer players).

#### Gaze Exploration Variability

The LMM revealed a significant fixed effect of trial cluster [*F*(2,58) = 839.330, *p* < 0.001, *η_*p*_^2^* = 0.97], but no significant fixed effect of group [*F*(1,29) = 2.124, *p* = 0.156, *η_*p*_^2^* = 0.07] and no significant interaction effect between trial cluster and group [*F*(2,58) = 0.949, *p* = 0.393, *η_*p*_^2^* = 0.03] on log transformed gaze exploration variability. The further analysis revealed (Figure 5C) a lower gaze exploration variability (*p* < 0.001) in cluster #2 (10.868 ± 1.373 degrees) than in cluster #1 (18.013 ± 2.330 degrees), a lower gaze exploration variability in cluster #3 (9.391 ± 1.012 degrees) than in clusters #1 and #2 (*p*s < 0.001).

#### Dwell Time

The LMM revealed a significant fixed effect of trial cluster [*F*(2, 521) = 7.792, *p* < 0.001, *η_*p*_^2^* = 0.03], a significant fixed effect of AOI [*F*(5,521) = 628,252, *p* < 0.001, *η_*p*_^2^* = 0.86], a two-way significant interaction effect between group and AOI [*F*(5,521) = 9.545, p < 0.001, *η_*p*_^2^* = 0.08], a two-way significant interaction effect between trial cluster and AOI [*F*(10,521) = 21.362, *p* < 0.001, *η_*p*_^2^* = 0.29], but no fixed effect of group [*F*(1,521) = 1.388, *p* = 0.239, *η_*p*_^2^* < 0.01], no two-way interaction effect between group and trial cluster [*F*(2,521) = 0.022, *p* = 0.978, *η_*p*_^2^* < 0.01], and finally no three-way interaction effect between group, trial cluster and AOI [*F*(10,521) = 1.805, *p* = 0.057, *η_*p*_^2^* = 0.03] on square-root-transformed dwell time.

The further analysis revealed (Figure 5D) a lower dwell time on centroid AOI (*p*s < 0.001) in trials of cluster #1 (0.318 ± 0.173 s) than in trials of clusters #2 (0.714 ± 0.325 s) and #3 (0.749 ± 0.232), but no difference between trials of clusters #2 and #3 (*p* = 0.823). For “other” AOI, it revealed higher dwell time (*p*s < 0.001) in trials of cluster #1 (4.478 ± 0.587 s) than in trials of clusters #2 (3.763 ± 0.730 s) and #3 (3.532 ± 0.459 s), but no difference between trials of clusters #2 and #3 (*p* = 0.445). For R1 AOI, the dwell time was lower (*p*s < 0.001) in trials of cluster #1 (1.296 ± 0.450 s) than in trials of clusters #2 (2.247 ± 0.884 s) and #3 (2.409 ± 0.632 s), but no difference was observed between trials of clusters #2 and #3 (*p* = 0.251). For R2 AOI, the dwell time was higher (*p* < 0.001) in trials of cluster #3 (1.804 ± 0.391 s) than in trials of cluster #1 (1.249 ± 0.444 s) and higher (*p* = 0.002) in trials of cluster #3 than in trials of cluster #2 (1.453 ± 0.553 s), but no difference was observed between trials of clusters #1 and #2 (*p* = 0.189). For R3 AOI, the dwell time was higher (*p* = 0.019) in trials of cluster #1 (1.311 ± 0.437 s) than in trials of cluster #3 (1.017 ± 0.232 s), but no difference (*p* = 0.239) was observed between trials of clusters #1 and #2 (1.150 ± 0.469 s) and between trials of clusters #2 and #3 (*p* = 0.521). For R4 AOI, the dwell time was higher (*p* = 0.017) in trials of cluster #1 (0.682 ± 0.292 s) than in trials of clusters #2 (0.480 ± 0.207 s) and higher (*p* < 0.001) in trials of cluster #1 than on trials of cluster #3 (0.402 ± 0.167 s), but no difference was observed between trials of clusters #2 and #3 (*p* = 0.461). Also, the further analysis revealed a higher dwell time on R1 AOI (*p* < 0.001) in soccer players (2.230 ± 0.846 s) than in non-soccer players (1.722 ± 0.743 s), a higher dwell time on R2 AOI (*p* = 0.002) in soccer players (1.638 ± 0.526 s) than in non-soccer players (1.357 ± 0.470), a lower dwell time on “other” AOI (*p* < 0.001) in soccer players (3.676 ± 0.733 s) than in non-soccer players (4.171 ± 0.617 s), but no difference between soccer players (1.194 ± 0.385 s) and non-soccer players (1.127 ± 0.430 s) on R3 AOI (*p* = 0.294), no difference between soccer players (0.488 ± 0.261 s) and non-soccer players (0.556 ± 0.246 s) on R4 AOI (*p* = 0.175) and no difference between soccer players (0.604 ± 0.305 s) and non-soccer players (0.584 ± 0.332 s) on centroid AOI (*p* = 0.601). Finally, for soccer players, the dwell time was higher for “other” AOI than for R1 AOI (*p* < 0.001), which was higher than R2 AOI (*p* < 0.001), which was higher than R3 AOI (*p* < 0.001), which was higher than R4 (*p* < 0.001), which was not different from centroid AOI (*p* = 0.305). For non-soccer players, the dwell time was higher for “other” AOI than for R1 AOI (*p* < 0.001), which was higher than R2 AOI (*p* < 0.001), which was not different from R3 AOI (*p* = 0.504), which was higher than R4 AOI (*p* < 0.001), which was not different from centroid AOI (*p* = 1.000).

#### Situation Score

The LMM revealed a significant fixed effect of trial cluster [*F*(2,58) = 26.520, *p* < 0.001, *η_*p*_^2^* = 0.48], a significant fixed effect of group [*F*(1,29) = 19.051, *p* < 0.001, *η_*p*_^2^* = 0.40], but no significant interaction effect [*F*(2,58) = 0.844, *p* = 0.435, *η_*p*_^2^* = 0.03] on mean situation score. The further analysis revealed (Figure 5E) a situation score lower (*p* < 0.001) in cluster #1 (2.804 ± 0.392) than in cluster #2 (3.242 ± 0.339), a situation score lower (*p* = 0.032) in cluster #1 than in cluster #3 (2.964 ± 0.430), and a situation score higher in cluster #2 than in cluster #3 (*p* < 0.001).

## Discussion

A multiple soccer players tracking task in a VR-HMD was proposed to study the ability of soccer players and non-soccer players to track multiple teammates and opponents as they would do on a real field. The investigation focused on both the influence that soccer-specific trajectories could have on visual tracking performance, but also the difference in gaze activity that could support the difference in tracking performance between soccer and non-soccer players.

### Visual Tracking Performance

In accordance with the literature (Faubert, 2013; Martín et al., 2017; Qiu et al., 2018; Harris et al., 2020a; Jin et al., 2020), visual tracking performance was higher in soccer players than in non-soccer players regardless of movement type. These findings supported the idea that soccer players have higher core perceptual-cognitive abilities like visual attention (Voss et al., 2010; Qiu et al., 2018; Jin et al., 2020) or working memory (Green and Bavelier, 2006; Harris et al., 2020a). Green and Bavelier observed that visual tracking performance may be enhanced after regular training with action video games (2006). In the same vein, regular practice in an environment as complex and dynamic as a soccer game may account for the greater ability of soccer players to gather information about multiple moving players on a virtual soccer field in a VR-HMD. However, findings did not support the expectation that soccer players would benefit from specific knowledge to track the multiple moving virtual players. Otherwise, the tracking performance between soccer and non-soccer players would have been greater when facing soccer-specific scenarios than when facing pseudo-random movement scenarios. It is likely that the proposed conditions did not require the use of soccer-specific knowledge to perform the visual tracking task. An alternative explanation for these results would be that contextualizing object movements to a domain of expertise simply does not influence visual tracking ability. The advantage of soccer players in this visual tracking task may be mainly related to the mechanisms underlying the acquisition of information about the dynamics of the scene, such as spatial attention and working memory (Ehmann et al., 2021), independent of its content.

### Gaze Activity

**FIGURE 6:**
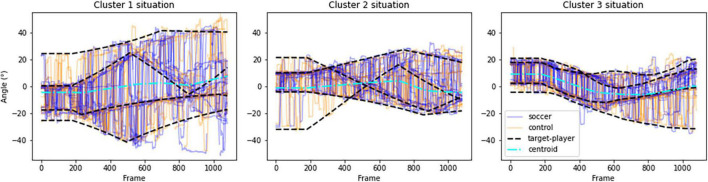
Gaze activity of all soccer players (blue lines) and all non-soccer players (orange lines) in three situations corresponding to cluster #1 (left plot), cluster #2 (center plot) and cluster #3 (right plot), respectively. The dashed black lines correspond to the target-players perceived direction and the dotted-dashed cyan lines correspond to target’s centroids perceived direction.

The proposed analysis grouped the situations according to the spatial distribution of virtual players into three clusters. As expected, some of the variability in gaze activity was explained by the spatial features of the situations presented. Figure 6 shows participants’ gaze angle (in blue for soccer players and in orange for non-soccer players) during the most typical trial in each of the three clusters, i.e., the trial closest to the center of each cluster. The high dispersion of target-players (black dashed lines in each plot of Figure 6) naturally induced larger saccades and visual exploration of participants in cluster #3 trials (Figure 6, left plot). Findings also revealed a higher search rate and a longer dwell time at fixing “other” AOI for situations of cluster #1 that were the most difficult for participants to complete. The consequent dispersion of target-players probably did not allow the use of an anchoring gaze strategy with enough visual acuity to monitor target-players in periphery. The repetition of long saccadic suppression of visual information likely affected tracking accuracy in situations from cluster #1 (Vater et al., 2017). Conversely, situations with lower target-players dispersion (clusters #2 and #3, center and right plots in Figure 6, respectively) induced a lower search rate, lower amplitude of saccades, a lower gaze exploration variability and higher dwell time on R1 and R2 AOIs. Using a lower search rate, gaze anchoring strategy and tracking virtual players with peripheral vision was more pronounced in these situations than in situations from cluster #1 (Vater et al., 2020). Furthermore, situation score of situations from cluster #3 was lower than situations from cluster #2, which confirmed the negative effect of crowding on visual tracking accuracy (Vater et al., 2017). Crowding increased the possibility to confuse a target-player with a disruptor. The cost of saccades likely also increased if several of the four target-players were crowded by nearby disruptors, which may have induced a further reduction in saccade amplitude and overall gaze exploration in cluster #3 situations compared to cluster #2 situations (Vater et al., 2020).

Search rate did not differ between soccer players and non-soccer players, which is consistent with findings of Harris et al. (2020a). However, gaze activity of soccer players and non-soccer players differed in two aspects. First, in situations with a wide dispersion of target-players (cluster #1), soccer players made saccades of greater amplitude than non-soccer players. Increasing amplitude of saccades in visual search seems to result from practice (Harris et al., 2021). Individuals learned to be more sensitive to peripheral cues and to make large saccades that land closer to the target in visual search tasks (Harris et al., 2021). Previous studies reported that soccer players are sensitive to visual cues located farther across the width of peripheral vision than non-football players (Hüttermann et al., 2014), probably due to regular practice requiring allocation of attention to the horizontal axis of peripheral vision (Hüttermann and Memmert, 2018). In this study, the soccer players likely benefited from extended attentional sensitivity that helped them make larger saccades during the tracking task in situations with spatially distant target-players. To confirm this assumption, further investigations are needed to see whether individuals with an extended attentional window (Hüttermann et al., 2014) would demonstrate better visual tracking accuracy in situations with spatially distant target-players and, conversely, whether the attentional window would be extended after training to track spatially distant target-players (Hüttermann and Memmert, 2018). Second, soccer players spent more time fixating the R1 and R2 target-players, i.e., the target-players closest to the centroid, and less time fixating the “other” AOI than non-soccer players. Very few gaze fixations were directed toward the centroid. Instead, the target-players closest to the centroid would be used as visual pivots to locate the gaze at a cost-optimized location in space between all target-players (Zelinsky and Neider, 2008; Oksama and Hyönä, 2016; Vater et al., 2020). In addition, for all participants, “other” was more fixated than any other AOI. It appears that visual pivot would not directly be on a specific target-player but rather on a location in the vicinity of that central target-player (Oksama and Hyönä, 2016). This shortcut would avoid the mental cost associated with continuously computing the actual centroid between target-players (Fehd and Seiffert, 2010; Oksama and Hyönä, 2016). The results revealed that soccer players executed this strategy more frequently than non-soccer players. They might have learnt this strategy from playing in complex and dynamic situations and it may have helped them in the proposed visual tracking task. To confirm this, future studies should investigate whether dwell time on “other” AOI decreases in favor of increased dwell time on central AOIs after regular soccer training.

Limitations regarding these findings on gaze activity have to be highlighted. First, the spatial threshold used to determine whether an AOI was fixed or not (1.5°) was chosen based on the registration error of the device (1.1°) and a margin related to the thickness of the virtual players (0.4°). The dwell time variable could have varied with another threshold value. These results need to be confirmed with another way of identifying fixed objects in the virtual environment. Instead of using a post-process calculation, it would be possible to record at each time step which object is fixated using a ray cast along the gaze vector in the virtual environment. Second, for simplicity, only two variables were used to cluster the situations, which may have retained some variability between trials within the same cluster. For example, some variability in search rate still existed within the same cluster of trials (Figure 5). Further investigations should consider other situational features, like speed (Harris et al., 2020a), to cluster the situations. At least, it would be interesting to complete this analysis by comparing the activity of the gaze with pre-selected situations based on the disparity of the target-players in space and the density of nearby disruptors.

### Perspectives

The results of this study open new perspectives to better appreciate the effect of expertise on visual tracking ability.

In this study, virtual players were modeled as simplified humanoid mannequins with no other discerning visual features because benefits to soccer players were expected with the simple use of structured relative movements of virtual players (North et al., 2016). However, using postural cues also helps soccer players anticipate the outcome of game situations (North et al., 2016). Prior studies have noted the higher ability of soccer players to perceive body kinematics compared to non-soccer players in soccer-specific or generic biological motion perception tasks (Romeas and Faubert, 2015) and that biological motion perception increased after regular MOT-based training (Legault and Faubert, 2012). Thus, when the implementation of virtual players with realistic body movements is possible, further research could observe the influence of postural cues on the visual tracking performance of soccer players.

Soccer players have to make important body and head rotations to collect information on the surrounding environment (McGuckian et al., 2018; Jordet et al., 2020; Aksum et al., 2021). The maximum dispersion between the leftmost and rightmost target-players was restricted to 90° in this study. This choice was made to allow participants to keep all four target-players in the field of view of the VR-HMD if they properly adjust their position and orientation to limit the difficulty of the tracking task. But ultimately, the extrapolation of locations would surely have been more important if, with a wider dispersion, all target-players could not be constantly visible. Facing a soccer-specific scenario, a soccer player is more likely than a non-soccer player to know where another player is according to playing positions and game logic, even if the player was not visible for seconds. But this advantage of soccer players would certainly disappear in pseudo-random scenarios because the virtual players would not follow any playing position or game logic in their trajectories. In future research, it would be wise to manipulate the dispersion of target-players in space to see if the advantage of soccer players is moderated by the need to extrapolate target-players who are outside the field of view. Moreover, with a wider dispersion among target-players, higher head or body excursion would be necessary (McGuckian et al., 2018; Aksum et al., 2021). Soccer players, who are accustomed to working with 360°constraints (Ehmann et al., 2021), would likely demonstrate different visual search rates than non-soccer players.

In a resource-based theory (Wickens, 2002), the number of targets that can be tracked depends on the available attentional resources (Tombu and Seiffert, 2008). Participants performed the tracking while standing, and were free to move around the given space. Simply standing and moving requires resources that are no longer available for the visual tracking task (see Woollacott and Shumway-Cook, 2002; Al-Yahya et al., 2011; Pothier et al., 2015 for reviews on global cognitive motor interference). Although we thought the proposed design was closer to the constraints of playing soccer than sitting in front of a screen, we assumed that it was still not sufficiently subject to the constraints of soccer multitasking. Indeed, soccer players have to collect information about their teammates and opponents while they interact, or will interact, with the ball (Jordet et al., 2020). The visual system and attentional resources are allocated to the interaction of the ball, which can generate interference with tracking teammates and opponents (Koch et al., 2018). Although the difference in average tracking performance between soccer and non-soccer players was not significantly greater when faced with soccer-specific scenarios in this study, it is still possible that soccer players provide less effort in the visual tracking task when facing soccer-specific scenarios. Pupillometry could be used in further investigations on sport-specific visual tracking tasks to control cognitive effort of participants (van der Wel and van Steenbergen, 2018; Cardoso et al., 2019). If the specific visual tracking task requires little cognitive effort for soccer players, then they would be able to perform a secondary task simultaneously. At least, visual tracking performance of soccer players would be more resistant to decline than that of non-soccer players when simultaneously performing a secondary task (Gabbett et al., 2011). The use of a motor-cognitive dual-task (see Schaefer, 2014 for a narrative review on expertise effect) should be considered in a future study to see if a greater difference in tracking performance between soccer and non-soccer players would emerge when acting in the environment during soccer-specific scenarios. Using a multitask paradigm with a secondary motor task, e.g., receiving a ball, would increase the representativeness of visual exploration conditions.

## Conclusion

The findings provide evidence that soccer players demonstrate higher ability than non-soccer players to track multiple virtual players in a VR-based soccer-specific environment. However, it was not possible in this study to observe an effect of the sport-specific nature of trajectories used to drive the virtual players. In addition, it was observed that gaze activity of participants varied according to the spatial distribution of virtual players, which also affected visual tracking performance. Furthermore, gaze activity varied between soccer and non-soccer players in some aspects, suggesting that visual tracking accuracy of soccer players was probably supported by more appropriate gaze strategies. Finally, future studies should consider a further step toward field constraints, such as adopting a dual-task methodology to better understand the relationship that may exist between sport performance and visual tracking performance.

## Data Availability Statement

The raw data supporting the conclusions of this article will be made available by the authors, without undue reservation.

## Ethics Statement

The studies involving human participants were reviewed and approved by the French National Ethics Committee CERSTAPS (IRB00012476-2021-16-04-102). The participants provided their written informed consent to participate in this study.

## Author Contributions

AV, AS, and RK contributed to the conception and design of the study. AV and AL performed the statistical analysis. AV wrote the first draft of the manuscript. All authors contributed to manuscript revision, read, and approved the submitted version.

## Conflict of Interest

The authors declare that the research was conducted in the absence of any commercial or financial relationships that could be construed as a potential conflict of interest.

## Publisher’s Note

All claims expressed in this article are solely those of the authors and do not necessarily represent those of their affiliated organizations, or those of the publisher, the editors and the reviewers. Any product that may be evaluated in this article, or claim that may be made by its manufacturer, is not guaranteed or endorsed by the publisher.

## Footnotes

1
https://www.vive.com/us/product/vive-pro-eye/specs/

2 STATS SportsVU; Available from: https://www.statsperform.com/team-performance/football-performance/optical-tracking/.
